# Supplementary material for: Feedback between a retinoid-related nuclear receptor and the let-7 microRNAs controls the pace and number of molting cycles in C. elegans
Source: eLife. 2022 Aug 15;11:e80010. doi: 10.7554/eLife.80010 (PMC9377799; doi:10.7554/eLife.80010)
Supplement: Supplementary file 1. — The active, lethargic, and wake-to-wake intervals are defined in the text. The values derived from longitudinal studies of stage-specific cohorts of singled, isogenic worms. The top row of each section corresponds to the same-day cohort of singled, wild-type worms. Dashes (–) beneath ‘RNAi’ indicate continuous cultivation of the worms on E. coli HT115(DE3). ‘N’ is the cumulative sample size from two independent trials. All p values were generated by pairwise comparisons between individual metrics tabulated for a specific cohort of test subjects and also for the same-day, age-matched cohort of control subjects: ****p ≤ 0.0001, ***p ≤ 0.001, *p ≤ 0.05, ordinary one-way ANOVA with Bonferroni’s correction for multiple comparisons. Entries in the top row of each subsection correspond to six distinct cohorts of control subjects. By order of first appearance in the table, the strains tested were N2, QK509 [let-7(n2853)], GR1395 [mgIs49], GR1436 [let-7(mg279)], ARF249 [let-7(mg279); mir-84(tm1304)], QK201 [let-7(xk41)], QK203 [let-7(xk44)], QK198 [let-7(xk39)], QK199 [let-7(xk42)], OP43 [wgIs43], ARF414 [nhr-23(aaa20)] and VT1066 [mir-48 mir-241(nDf51); mir-84(n4037)]. Notably, both QK509 [let-7(n2853)] and the ancestral strain MT7626 [let-7(n2853)] developed at an accelerated pace: 71% of QK059 hatchlings and 79% of MT7626 hatchlings transited the larval stages and emerged as young adults within 42 h of cultivation with food, as compared with 12% of N2 hatchlings (N = 100, p ≤ 0.0001, chi-square test). [file elife-80010-supp1.docx]

**Supplementary File 1 – Relates to Figures 2, 4 and 6**

| Metrics of the Molting Biorhythm Associated with Specific Genotypes | | | | | | | | | | | | | | | |
| --- | --- | --- | --- | --- | --- | --- | --- | --- | --- | --- | --- | --- | --- | --- | --- |
|  | | | Interval (h) | | | | | | | | | | | |  |
| L4 stage cohort | | | Active | | | | Lethargic | | | | Wake-to-Wake | | | |  |
| Strain | RNAi | N | mean | sd | cv | p | mean | sd | cv | p | mean | sd | cv | p |  |
| wild type (N2) | － | 16 | 8.1 | 0.5 | 0.06 | － | 2.2 | 0.4 | 0.18 | － | 10.3 | 0.4 | 0.05 | － |  |
| wild type | *nhr-23* | 17 | 8.4 | 0.8 | 0.09 | n.s. | 4.6 | 0.7 | 0.16 | **** | 13.0 | 1.1 | 0.08 | **** |  |
| *let-7(n2853)* | － | 15 | 6.3 | 0.4 | 0.08 | **** | 1.5 | 0.5 | 0.33 | * | 7.9 | 0.6 | 0.08 | **** |  |
| *let-7(n2853)* | *nhr-23* | 19 | 6.7 | 0.6 | 0.10 | **** | 3.9 | 0.6 | 0.16 | **** | 10.6 | 0.8 | 0.07 | n.s. |  |
| *let-7(n2853)*† | － | 17 | 6.7 | 0.6 | 0.09 | **** | 1.6 | 0.5 | 0.30 | ** | 8.4 | 0.6 | 0.07 | **** |  |
| wild type (GR1395) | **－** | 20 | 7.8 | 0.5 | 0.07 | **－** | 2.2 | 0.6 | 0.18 | **－** | 10.0 | 0.5 | 0.05 | **－** |  |
| *let-7(mg279)* | **－** | 20 | 7.2 | 0.6 | 0.08 | ** | 2.2 | 0.4 | 0.17 | n.s. | 9.3 | 0.6 | 0.06 | ** |  |
| *let-7(mg279) mir-84(tm1304)* | **－** | 18 | 6.1 | 0.9 | 0.15 | **** | 2.4 | 0.6 | 0.25 | n.s. | 8.5 | 0.9 | 0.15 | **** |  |
| wild type (N2) | － | 17 | 7.7 | 0.5 | 0.07 | － | 2.0 | 0.0 | 0.00 | － | 9.7 | 0.5 | 0.05 | － | －^§^ |
| *let-7(xk41-scRORE1,2)* | **－** | 12 | 6.9 | 0.7 | 0.10 | * | 1.8 | 0.6 | 0.36 | n.s. | 8.7 | 0.9 | 0.10 | *** | *** |
| *let-7(xk44-scRORE1,2)* | － | 8 | 6.6 | 0.5 | 0.08 | ** | 2.3 | 0.5 | 0.21 | n.s. | 8.9 | 0.4 | 0.04 | * | ** |
| *let-7(xk39-scRORE1,3)* | **－** | 17 | 6.5 | 0.6 | 0.10 | **** | 2.4 | 0.6 | 0.26 | n.s. | 8.7 | 0.6 | 0.07 | **** | **** |
| *let-7(xk42-scRORE1,3)* | － | 14 | 6.6 | 1.1 | 0.17 | *** | 2.1 | 0.6 | 0.30 | n.s. | 8.6 | 0.7 | 0.09 | **** | **** |
| wild type (N2) | － | 12 | 8.1 | 0.7 | 0.08 | － | 2.0 | 0.0 | 0.0 | － | 10.0 | 0.7 | 0.07 | － |  |
| *wgIs43 [nhr-23++]* | **－** | 17 | 7.2 | 0.5 | 0.07 | *** | 2.2 | 0.6 | 0.29 | n.s. | 9.4 | 0.5 | 0.05 | ** |  |
| *nhr-23* (*aaa20-∆LCS*) | **－** | 25 | 6.8 | 0.7 | 0.1 | **** | 2.1 | 0.3 | 0.16 | n.s. | 8.9 | 0.6 | 0.07 | **** |  |
| L3 stage cohort | | | Active | | | | Lethargic | | | | Wake-to-Wake | | | |  |
| Strain | RNAi | N | mean | sd | cv | p | mean | sd | cv | p | mean | sd | cv | p |  |
| wild type (N2) | － | 17 | 6.3 | 0.4 | 0.07 | － | 1.6 | 0.5 | 0.31 | － | 7.9 | 0.7 | 0.08 | － |  |
| wild type | *nhr-23* | 12 | 7.7 | 1.2 | 0.15 | **** | 3.8 | 1.9 | 0.49 | **** | 11.0‡ | 1.3 | 0.12 | **** |  |
| *let-7(n2853)* | － | 18 | 5.6 | 0.6 | 0.11 | * | 1.3 | 0.4 | 0.36 | n.s. | 6.8 | 0.5 | 0.07 | * |  |
| *let-7(n2853)* | *nhr-23* | 18 | 5.8 | 0.8 | 0.13 | n.s. | 3.8 | 0.6 | 0.17 | **** | 9.6 | 0.9 | 0.09 | **** |  |
| wild type (N2) | － | 13 | 6.4 | 0.7 | 0.11 | － | 1.4 | 0.5 | 0.37 | － | 7.8 | 0.6 | 0.08 | － |  |
| *wgIs43 [nhr-23++]* | **－** | 15 | 5.2 | 0.9 | 0.18 | *** | 1.7 | 0.6 | 0.34 | n.s. | 6.9 | 0.6 | 0.09 | ** |  |
| *nhr-23* (*aaa20-∆LCS*) | **－** | 19 | 5.1 | 0.7 | 0.14 | *** | 1.2 | 0.5 | 0.44 | n.s. | 6.3 | 0.7 | 0.11 | **** |  |
| L2 stage cohort | | | Active | | | | Lethargic | | | | Wake-to-Wake | | | |  |
| Strain | RNAi | N | mean | sd | cv | p | mean | sd | cv | p | mean | sd | cv | p |  |
| wild type (N2) | － | 18 | 5.9 | 0.3 | 0.05 | **－** | 1.3 | 0.5 | 0.36 | **－** | 7.2 | 0.4 | 0.06 | **－** |  |
| wild type | *nhr-23* | 19 | 6.1 | 0.6 | 0.10 | n.s. | 3.9 | 1.1 | 0.28 | **** | 9.9 | 1.0 | 0.10 | **** |  |
| *mir-48 mir-241 (nDf51); mir-84(n4037)* | － | 17 | 6.3 | 0.7 | 0.11 | n.s. | 1.3 | 0.5 | 0.36 | n.s. | 7.6 | 0.7 | 0.10 | n.s. |  |
| *mir-48 mir-241 (nDf51); mir-84(n4037)* | *nhr-23* | 15 | 6.4 | 0.8 | 0.13 | n.s. | 3.0 | 0.4 | 0.13 | **** | 9.4 | 0.8 | 0.09 | **** |  |
| †Entry for the L3 cohort fortuitously observed throughout L4 and depicted by the penultimate actogram in Figure 2C. | | | | | | | | | | | | | | |  |
| ‡Value excludes the one and only *nhr-23(RNAi)* larvae that remained lethargic at the final time-sample. | | | | | | | | | | | | | | |  |
| § p-values of wake-to-wake interval by Mann-Whitney Test | | | | | | | | | | | | | | |  |
