# Supplementary material for: Feedback between a retinoid-related nuclear receptor and the let-7 microRNAs controls the pace and number of molting cycles in C. elegans
Source: eLife. 2022 Aug 15;11:e80010. doi: 10.7554/eLife.80010 (PMC9377799; doi:10.7554/eLife.80010)
Supplement: Supplementary file 2. — Entries correspond to sites shown in Figure 10D. The number of nucleotides between the 3′ end of each LCS and the stop codon is indicated. The thermostability of every RNA duplex between a prospective LCS and mature let-7, as predicted by RNAhybrid, was lower than the predicted thermostability (−29 kcal/mol) of duplexes between the functional LCS in the 3′ UTR of lin-41 and let-7 (Rehmsmeier et al., 2004). The 3′ UTRs were supported by ESTs archived in WBcel235/ce11, WBPS9, GRCh38/hg38, GRCm38/mm10, and GRCz10/danRer10. [file elife-80010-supp2.docx]

**Supplementary File 2 – Relates to Figures 5 and 10**

| *let-7* Consensus Sites (LCSs) Identified in 3' UTRs of Selected Nematode and Vertebrate Homologs of *ROR* | | | | | | |
| --- | --- | --- | --- | --- | --- | --- |
| Species | Gene | Identifier | 3' UTR Length  (nt.) | LCS  Position  (3' nt.) | TS  (kcal/mol) | Alignment of LCS (5' to 3') with *let‑7* (3' to 5') |
| *C. elegans* | *nhr-23* | NM_001025806 | 868 | 42 | -21.8 | 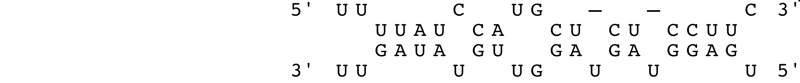 |
|  |  |  |  | 249 | -15.2 | 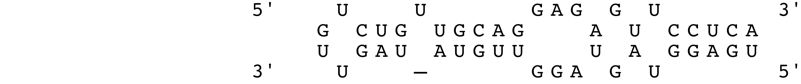 |
|  |  |  |  | **594** | -17.0 | 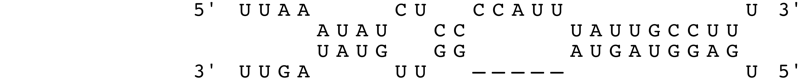 |
|  |  |  |  | 646 | -17.6 | 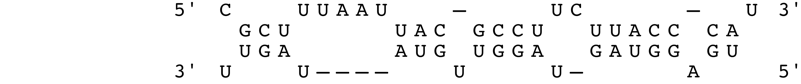 |
| *C. briggsae* | *nhr-23* | WBGene  00040598 | 866 | 629 | -20.7 | 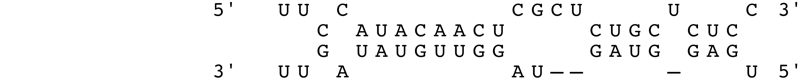 |
|  |  |  |  | **835** | -21.8 | 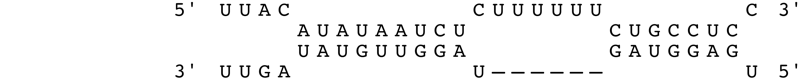 |
| *H. sapiens* | *RORβ* | NM_006914 | 7559 | **3576** | -25.9 | 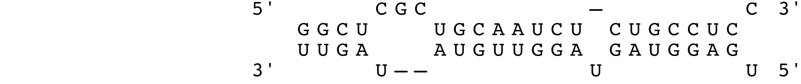 |
|  |  |  |  | **4055** | -23.1 | 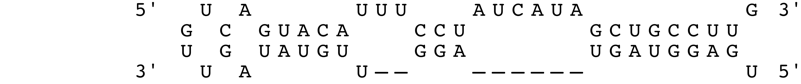 |
|  |  |  |  | 4961 | -23.1 | 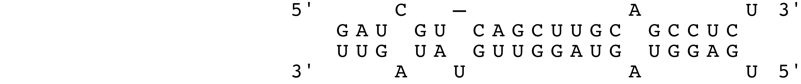 |
| *M. musculus* | *RORβ* | NM_146095 | 7271 | **3817** | -23.8 | 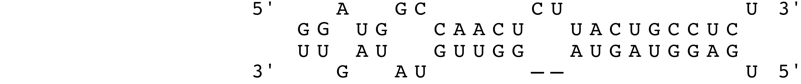 |
|  |  |  |  | 5242 | -22.7 | 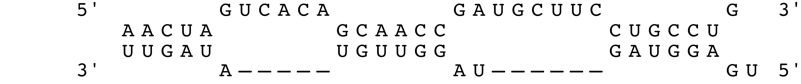 |
|  |  |  |  | 6675 | -26.7 | 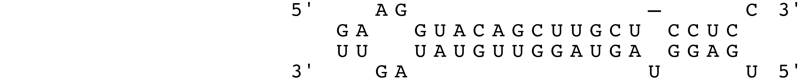 |
| *D. rerio* | *RORβ* | NM_001082856 | 5431 | **949** | -21.8 | 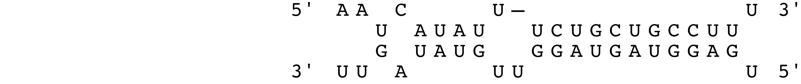 |
|  |  |  |  | **4318** | -23.8 | 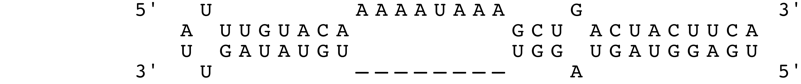 |
|  |  |  |  | **4421** | -23.3 | 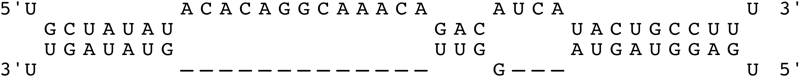 |
| *H. sapiens* | *RORα* | NM_134261 | 9171 | 3079 | -23.7 | 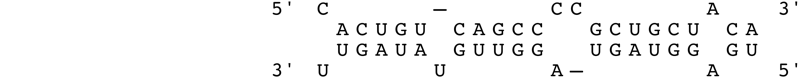 |
|  |  |  |  | 3142 | -22.8 | 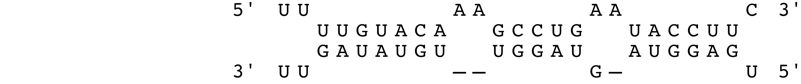 |
|  |  |  |  | 6480 | -24.0 | 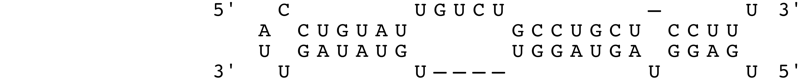 |
|  |  |  |  | **8321** | -22.2 | 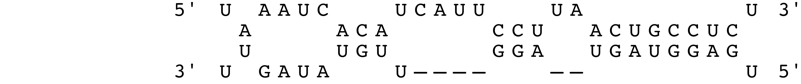 |
| *M. musculus* | *RORα* | NM_013646 | 9285 | **2055** | -23.9 | 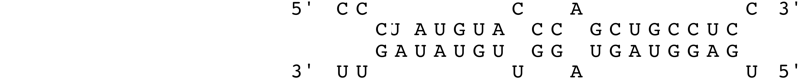 |
|  |  |  |  | **2184** | -23.7 | 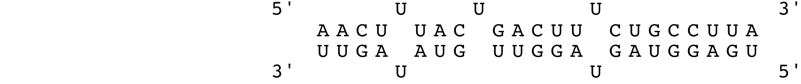 |
|  |  |  |  | 4913 | -22.6 | 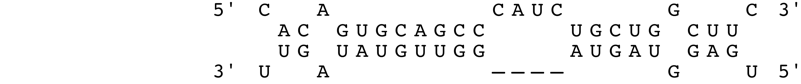 |
