# Supplementary material for: Feedback between a retinoid-related nuclear receptor and the let-7 microRNAs controls the pace and number of molting cycles in C. elegans
Source: eLife. 2022 Aug 15;11:e80010. doi: 10.7554/eLife.80010 (PMC9377799; doi:10.7554/eLife.80010)
Supplement: Supplementary file 3. — As described in Materials and methods, Metacycle was used to calculate the amplitude and phase of expression of the waveforms. The peak values and the slope of rise and decay were obtained by manual calculation. [file elife-80010-supp3.docx]

**Supplementary File 3 – Relates to Figures 5, 7, 8**

| **Comparisons among wild type, *nhr-23*** ***(∆LCS)* and *let-7(n2853)*** | | | | | | | | | | | | | |
| --- | --- | --- | --- | --- | --- | --- | --- | --- | --- | --- | --- | --- | --- |
| **Transcript:** | ***nhr-23*** | | | | | | | | | | | | |
|  |  |  | | | |  | | |  | | |  |  |
|  | **Replicate 1** | | | | | | | | | | | | |
| Stage | L3 | | | | | | | | | L4 | | | |
| Genotype | wild type | *nhr-23 (∆LCS)* | | | | wild type | | | *let-7 (n2853)* | wild type | *nhr-23 (∆LCS)* | wild type | *let-7 (n2853)* |
| Amplitude | 0.4 | 1.1 | | | | 0.5 | | | 0.9 | 0.3 | 0.4 | 0.1 | 0.8 |
| Phase | 3.2 | 2.4 | | | | 3.2 | | | 3.0 | 0.4 | 3.2 | 0.04 | 3.8 |
| Rising Slope | 0.19 | 0.46 | | | | 0.15 | | | 0.71 | 0.19 | 0.21 | 0.11 | 0.26 |
| Falling Slope | 0.50 | 0.49 | | | | 0.36 | | | 0.45 | 0.21 | 0.19 | 0.20 | 0.38 |
| Peak Value | 1.78 | 2.93 | | | | 1.69 | | | 2.84 | 1.14 | 1.81 | 1.18 | 2.65 |
|  |  |  | | | |  | | |  |  |  |  |  |
|  | **Replicate 2** | | | | | | | | | | | | |
| Stage | L3 | | | | | | | | | L4 | | | |
| Genotype | wild type | *nhr-23 (∆LCS)* | | | | wild type | | | *let-7*  *(n2853)* | wild type | *nhr-23*  *(∆LCS)* | wild type | *let-7*  *(n2853)* |
| Amplitude | 0.3 | 0.4 | | | | 0.2 | | | 0.3 | 0.2 | 0.7 | 0.1 | 0.3 |
| Phase | 3.1 | 2.1 | | | | 4.5 | | | 2.0 | 5.3 | 4.0 | 4.5 | 4.8 |
| Rising Slope | 0.12 | 0.30 | | | | 0.17 | | | 0.33 | 0.11 | 0.40 | 0.05 | 0.25 |
| Falling Slope | 0.14 | 0.17 | | | | 0.10 | | | 0.14 | 0.24 | 0.39 | 0.12 | 0.19 |
| Peak Value | 1.31 | 1.57 | | | | 1.17 | | | 2.02 | 1.30 | 2.43 | 0.94 | 2.25 |
|  |  |  | | | |  | | |  |  |  |  |  |
| **Transcript:** | ***fbn-1*** | | | | | | | | | | |  |  |
|  |  |  | | | |  | | |  |  |  |  |  |
|  | **Replicate 1** | | | | | | | | | | |  |  |
| Stage | L3 | | | | | | | | L4 | | |  |  |
| Genotype | wild type | *nhr-23*  *(∆LCS)* | | | | *let-7*  *(n2853)* | | | wild  type | *nhr-23*  *(∆LCS)* | *let-7*  *(n2853)* |  |  |
| Amplitude | 1.4 | 3.0 | | | | 2.1 | | | 1.2 | 1.7 | 1.9 |  |  |
| Phase | 2.1 | 2.4 | | | | 2.8 | | | 3.6 | 2.9 | 2.9 |  |  |
| Rising Slope | 0.73 | 1.56 | | | | 1.85 | | | 0.78 | 1.45 | 2.35 |  |  |
| Falling Slope | 0.64 | 1.59 | | | | 1.02 | | | 0.60 | 1.00 | 0.91 |  |  |
| Peak Value | 3.65 | 7.15 | | | | 5.19 | | | 4.24 | 6.63 | 5.94 |  |  |
|  |  |  | | | |  | | |  |  |  |  |  |
|  | **Replicate 2** | | | | | | | | | | |  |  |
| Stage | L3 | | | | | | | | L4 | | |  |  |
| Genotype | wild type | *nhr-23*  *(∆LCS)* | | | | *let-7*  *(n2853)* | | | wild  type | *nhr-23*  *(∆LCS)* | *let-7*  *(n2853)* |  |  |
| Amplitude | 0.7 | 0.6 | | | | 0.5 | | | 0.6 | 0.8 | 0.7 |  |  |
| Phase | 2.2 | 1.5 | | | | 1.6 | | | 6.3 | 4.6 | 5.2 |  |  |
| Rising Slope | 0.33 | 0.33 | | | | 1.10 | | | 0.30 | 0.37 | 0.20 |  |  |
| Falling Slope | 0.70 | 0.70 | | | | 0.50 | | | 0.40 | 1.35 | 0.28 |  |  |
| Peak Value | 1.90 | 2.10 | | | | 1.70 | | | 2.00 | 2.90 | 1.50 |  |  |
|  |  |  | | | |  | | |  |  |  |  |  |
| **Transcript:** | ***mlt-10*** | | | | | | | | | | |  |  |
|  |  |  | | | |  | | |  |  |  |  |  |
|  | **Replicate 1** | | | | | | | | | | |  |  |
| Stage | L3 | | | | | | | | L4 | | |  |  |
| Genotype | wild type | *nhr-23*  *(∆LCS)* | | | | *let-7*  *(n2853)* | | | wild  type | *nhr-23*  *(∆LCS)* | *let-7*  *(n2853)* |  |  |
| Amplitude | 1.0 | 2.3 | | | | 2.2 | | | 1.0 | 2.8 | 2.4 |  |  |
| Phase | 5.4 | 4.9 | | | | 5.1 | | | 6.2 | 5.0 | 4.9 |  |  |
| Rising Slope | 0.46 | 1.95 | | | | 1.76 | | | 0.51 | 1.91 | 2.10 |  |  |
| Falling Slope | 0.88 | 0.95 | | | | 0.65 | | | 0.30 | 0.69 | 0.63 |  |  |
| Peak Value | 3.95 | 4.36 | | | | 4.56 | | | 1.92 | 4.31 | 5.17 |  |  |
|  |  |  | | | | | | | |  |  |  |  |
|  | **Replicate 2** | | | | | | | | | | |  |  |
| Stage | L3 | | | | | | | | L4 | | |  |  |
| Genotype | wild type | *nhr-23*  *(∆LCS)* | | | | *let-7*  *(n2853)* | | | wild  type | *nhr-23*  *(∆LCS)* | *let-7*  *(n2853)* |  |  |
| Amplitude | 0.8 | 0.9 | | | | 0.2 | | | 0.8 | 1.0 | 0.6 |  |  |
| Phase | 2.7 | 1.5 | | | | 2.8 | | | 8.6 | 6.6 | 6.3 |  |  |
| Rising Slope | 0.28 | 0.58 | | | | 0.40 | | | 0.37 | 0.45 | 0.38 |  |  |
| Falling Slope | 0.60 | 0.60 | | | | 0.23 | | | 0.38 | 0.40 | 0.35 |  |  |
| Peak Value | 1.80 | 3.20 | | | | 1.90 | | | 2.80 | 2.60 | 2.50 |  |  |
|  |  |  | | | |  | | |  |  |  |  |  |
| **Transcript:** | ***lin-42*** | | | | | | | | | | |  |  |
|  |  |  | | | |  | | |  |  |  |  |  |
|  | **Replicate 1** | | | | | | | | | | |  |  |
| Stage | L3 | | | | | | | | L4 | | |  |  |
| Genotype | wild type | *nhr-23*  *(∆LCS)* | | | | *let-7*  *(n2853)* | | | wild  type | *nhr-23*  *(∆LCS)* | *let-7*  *(n2853)* |  |  |
| Amplitude | 0.6 | 1.7 | | | | 1.5 | | | 0.8 | 1.6 | 1.2 |  |  |
| Phase | 4.5 | 3.9 | | | | 4.2 | | | 5.4 | 4.8 | 4.7 |  |  |
| Rising Slope | 0.20 | 0.93 | | | | 0.78 | | | 0.27 | 0.70 | 0.53 |  |  |
| Falling Slope | 0.30 | 0.86 | | | | 0.78 | | | 0.37 | 0.52 | 0.44 |  |  |
| Peak Value | 2.12 | 4.15 | | | | 3.88 | | | 2.50 | 3.48 | 2.94 |  |  |
|  |  |  | | | |  | | |  |  |  |  |  |
|  | **Replicate 2** | | | | | | | | | | |  |  |
| Stage | L3 | | | | | | | | L4 | | |  |  |
| Genotype | wild type | | *nhr-23*  *(∆LCS)* | | | | *let-7*  *(n2853)* | | wild type | *nhr-23*  *(∆LCS)* | *let-7*  *(n2853)* |  |  |
| Amplitude | 0.4 | | 0.6 | | | | - | | 0.6 | 0.8 | 0.7 |  |  |
| Phase | 1.9 | | 0.3 | | | | - | | 6.20 | 4.10 | 5.00 |  |  |
| Rising Slope | 0.33 | | 0.80 | | | | - | | 0.20 | 0.80 | 0.20 |  |  |
| Falling Slope | 0.48 | | 0.30 | | | | - | | 0.65 | 0.28 | 0.30 |  |  |
| Peak Value | 2.30 | | 2.50 | | | | - | | 2.00 | 2.30 | 2.10 |  |  |
|  |  |  | | | |  | | |  | | |  |  |
| **Comparisons between vector and *nhr-23(RNAi)*** | | | | | | | | | |  |  |  |  |
| **Transcript:** | ***fbn-1*** | | | | | | | | |  |  |  |  |
|  |  | | |  | | |  | |  |  |  |  |  |
| Stage | L3 | | | | | | L4 | | |  |  |  |  |
| RNAi | mock | | | *nhr-23*  *(RNAi)* | | | mock | | *nhr-23*  *(RNAi)* |  |  |  |  |
| Amplitude | 0.7 | | | 0.2 | | | 1.0 | | 0.2 |  |  |  |  |
| Phase | 2.0 | | | 2.0 | | | 1.9 | | 2.1 |  |  |  |  |
| Rising Slope | 0.68 | | | 0.14 | | | 0.40 | | 0.30 |  |  |  |  |
| Falling Slope | 0.21 | | | 0.15 | | | 0.42 | | 0.12 |  |  |  |  |
| Peak Value | 1.85 | | | 0.90 | | | 2.80 | | 1.20 |  |  |  |  |
|  |  | | |  | | |  | |  |  |  |  |  |
| **Transcript:** | ***mlt-10*** | | | | | | | | |  |  |  |  |
|  |  | | |  | | |  | |  |  |  |  |  |
| Stage | L3 | | | | | | L4 | | |  |  |  |  |
| RNAi | mock | | | *nhr-23*  *(RNAi)* | | | mock | | *nhr-23*  *(RNAi)* |  |  |  |  |
| Amplitude | 0.7 | | | 0.5 | | | 0.7 | | 0.5 |  |  |  |  |
| Phase | 5.9 | | | 6.1 | | | 4.9 | | 4.9 |  |  |  |  |
| Rising Slope | 0.36 | | | 0.08 | | | 0.40 | | 0.10 |  |  |  |  |
| Falling Slope | 0.58 | | | 0.20 | | | 0.33 | | 0.15 |  |  |  |  |
| Peak Value | 2.10 | | | 0.80 | | | 1.90 | | 0.80 |  |  |  |  |
|  |  | | |  | | |  | |  |  |  |  |  |
| **Transcript:** | ***lin-42*** | | | | | | | | |  |  |  |  |
|  |  | | |  | | |  | |  |  |  |  |  |
| Stage | L3 | | | | | | L4 | | |  |  |  |  |
| RNAi | mock | | | *nhr-23*  *(RNAi)* | | | mock | | *nhr-23*  *(RNAi)* |  |  |  |  |
| Amplitude | 0.7 | | | 0.5 | | | 0.6 | | 0.2 |  |  |  |  |
| Phase | 3.2 | | | 5.3 | | | 4.8 | | 4.1 |  |  |  |  |
| Rising Slope | 0.35 | | | 0.15 | | | 0.60 | | 0.10 |  |  |  |  |
| Falling Slope | 0.33 | | | 0.25 | | | 0.50 | | 0.15 |  |  |  |  |
| Peak Value | 2.10 | | | 1.30 | | | 1.80 | | 0.70 |  |  |  |  |
|  |  | | |  | | |  | |  |  |  |  |  |
| **Comparisons among wild type, *nhr-23(aaa20)* and *mir-48 mir-241(nDf51); mir-84(n4037)*** | | | | | | | | |  |  |  |  |  |
| **Transcript:** | ***nhr-23*** | | | | | | | |  |  |  |  |  |
|  |  | | |  | | |  | |  |  |  |  |  |
| Stage | L2 | | | | | | | |  |  |  |  |  |
| Genotype | wild type | | | | *nhr-23 (∆LCS)* | | | *mir-48  mir-241*  *(nDf51);  mir-84*  *(n4037)* |  |  |  |  |  |
| Amplitude | 0.7 | | | | 1.6 | | | 0.6 |  |  |  |  |  |
| Phase | 1.8 | | | | 2.0 | | | 1.6 |  |  |  |  |  |
| Rising Slope | 0.38 | | | | 1.64 | | | 1.10 |  |  |  |  |  |
| Falling Slope | 0.39 | | | | 2.00 | | | 0.21 |  |  |  |  |  |
| Peak Value | 2.20 | | | | 5.10 | | | 2.60 |  |  |  |  |  |
|  |  | | | |  | | |  |  |  |  |  |  |
