# Supplementary material for: Feedback between a retinoid-related nuclear receptor and the let-7 microRNAs controls the pace and number of molting cycles in C. elegans
Source: eLife. 2022 Aug 15;11:e80010. doi: 10.7554/eLife.80010 (PMC9377799; doi:10.7554/eLife.80010)
Supplement: Supplementary file 4. — The bioinformatic approaches and criteria for assignment of queries to categories are described in Materials and methods. The name and WormBase accession number of each gene are listed. ‘# Obs./# Exp.’ stands for the number of observed DNA or RNA response elements divided by the number of elements predicted by chance alone. The down arrows denote downregulation of the query transcript in nhr-23(RNAi) animals as compared with wild-type controls. The ‘+’ symbol in column 12 denotes identification of the transcript in ALG-1 iCLIP datasets in vivo (Broughton et al., 2016). The symbol ‘♒’ indicates that expression of the gene oscillates across larval development. Relevant datasets are identified in the text, Materials and Methods, and Key Resources Table. [file elife-80010-supp4.docx]

**Supplementary File 4 – relates to Figures 8 and 9**

| Classification of Clock-Controlled Genes (CCGs) as Direct Targets of NHR-23, *let-7s*, neither or both. | | | | | | | | | | | | | | |
| --- | --- | --- | --- | --- | --- | --- | --- | --- | --- | --- | --- | --- | --- | --- |
| Gene Name | Sequence | Criteria for targets of NHR-23: | | | | | NHR-23 target (Y/N) | Criteria for targets of *let-7s*: | | | | *let-7s* target (Y/N) | Target class | Cyclic mRNA levels (♒) |
|  |  | Size of upstream regulatory region & first intron (kb) | ROREs | | mRNA levels after  *nhr-23* RNAi | NHR-23 ChIP-Seq Peaks (#) |  | Size of 3' UTR (nt.) | LCSs | | ALG-1 iCLIP Peaks (+/-) |  |  |  |
|  |  |  | # | # Obs. # Exp. |  |  |  |  | # | # Obs. # Exp. |  |  |  |  |
|  |  |  |  |  |  |  |  |  |  |  |  |  |  |  |
|  |  |  |  |  |  |  |  |  |  |  |  |  |  |  |
|  |  |  |  |  |  |  |  |  |  |  |  |  |  |  |
|  |  |  |  |  |  |  |  |  |  |  |  |  |  |  |
| ***Potential Key* *Clock Components*** | | | | | | | | | | | | | | |
| *let-7* | C05G5.6 | 1.5 | 3 | 5.8 | ⬇ | 1 | Y | N/A | – | – | – | N/A | NHR-23 | ♒ |
| *lin-42a* | F47F6.1 | 3.7 | 3 | 2.3 | ⬇ | 4 | Y | 939 | 4 | 3.2 | **＋** | Y | Shared | ♒ |
| *lin-42b* | F47F6.1 | 5.5 | 3 | 1.6 | ⬇ | 3 | Y | 939 | 4 | 3.2 | **＋** | Y | Shared | ♒ |
| *lin-42c* | F47F6.1 | 5.5 | 3 | 1.6 | ⬇ | 3 | Y | 156 | 0 | 0.0 | – | N | NHR-23 | ♒ |
| *mir-48* | F56A12.3 | 1.7 | 2 | 3.4 | – | 1 | Y | N/A | – | – | – | N/A | NHR-23 | ♒ |
| *mir-241* | F56A12.4 | 2.0 | 2 | 2.9 | – | 2 | Y | N/A | – | – | – | N/A | NHR-23 | ♒ |
| *mir-84* | B0395.4 | 2.8 | 1 | 1.1 | – | 2 | Y | N/A | – | – | – | N/A | NHR-23 | ♒ |
| *nhr-23* | C01H6.5 | 6.1 | 8 | 3.8 | ⬇ | 3 | Y | 868 | 4 | 2.6 | **＋** | Y | Shared | ♒ |
| *nhr-25* | F11C1.6 | 6.9 | 3 | 1.3 | – | 3 | Y | 749 | 1 | 1.0 | **＋** | Y | Shared | ♒ |
| ***Other Gene Regulatory Factors*** | | | | | | | | | | | | | | |
| *alg-1* | F48F7.1 | 9.9 | 11 | 3.2 | – | 3 | Y | 400 | 1 | 1.9 | **＋** | Y | Shared | ♒ |
| *bed-3* | F25H8.6 | 1.7 | 2 | 3.4 | – | 1 | Y | 459 | 1 | 1.6 | **＋** | Y | Shared | ♒ |
| *blmp-1* | F25D7.3 | 6.7 | 7 | 3.0 | – | 4 | Y | 861 | 3 | 2.6 | **＋** | Y | Shared | ♒ |
| *bro-1* | F56A3.5 | 1.2 | 0 | 0.0 | – | 1 | N | 379 | 1 | 2.0 | – | N | **–** | ♒ |
| *dre-1* | K04A8.6 | 7.5 | 8 | 3.1 | – | 4 | Y | 376 | 2 | 4.0 | **＋** | Y | Shared | ♒ |
| *mab-10* | R166.1 | 6.0 | 8 | 3.9 | – | 2 | Y | 374 | 1 | 2.0 | – | N | NHR-23 | ♒ |
| *nhr-41* | Y104H12A.1 | 11.1 | 23 | 6.0 | – | 2 | Y | 332 | 1 | 2.3 | – | N | NHR-23 | ♒ |
| *pqn-47* | F59B10.1 | 5.9 | 7 | 3.4 | – | 5 | Y | 804 | 2 | 1.9 | **＋** | Y | Shared | ♒ |
| *rnt-1* | B0414.2 | 9.2 | 4 | 1.3 | – | 0 | N | 221 | 0 | 0.0 | – | N | **–** | ♒ |
| ***Signaling Pathway Components*** | | | | | | | | | | | | | | |
| *acn-1* | C42D8.5 | 4.0 | 4 | 2.9 | ⬇ | 3 | Y | 384 | 1 | 2.0 | **＋** | Y | Shared | ♒ |
| *apl-1* | C42D8.8 | 4.9 | 4 | 2.4 | – | 5 | Y | 678 | 1 | 1.1 | **＋** | Y | Shared | ♒ |
| *calu-1* | M03F4.7 | 1.7 | 3 | 5.1 | – | 3 | Y | 256 | 1 | 3.0 | **＋** | Y | Shared | ♒ |
| *cki-1* | T05A6.1 | 1.9 | 2 | 3.1 | – | 1 | Y | 235 | 2 | 6.5 | **＋** | Y | Shared | ♒ |
| *glf-1* | H04M03.4 | 2.4 | 1 | 1.2 | ⬇ | 1 | Y | 247 | 2 | 6.2 | – | N | NHR-23 | ♒ |
| *lon-1* | F48E8.1 | 4.8 | 3 | 1.8 | – | 5 | Y | 185 | 2 | 8.3 | **＋** | Y | Shared | ♒ |
| *lrp-1* | F29D11.1 | 7.9 | 3 | 1.1 | – | 6 | Y | 346 | 2 | 4.4 | **＋** | Y | Shared | ♒ |
| *mlt-8* | W08F4.6 | 3.5 | 2 | 1.7 | ⬇ | 1 | Y | 270 | 2 | 5.6 | **＋** | Y | Shared | ♒ |
| *nekl-2* | ZC581.1 | 1.1 | 1 | 2.6 | – | 0 | N | 73 | 0 | 0.0 | – | N | **–** | ♒ |
| *nlp-22* | T24D8.3 | 0.8 | 0 | 0.0 | – | 0 | N | 1000 | 2 | 1.5 | – | N | **–** | ♒ |
| *osm-7* | T05D4.4 | 3.5 | 1 | 0.8 | – | 0 | N | 121 | 1 | 6.5 | **＋** | Y | *let-7s* | ♒ |
| *osm-11* | F11C7.5 | 2.8 | 1 | 1.1 | – | 3 | Y | 545 | 4 | 5.5 | **＋** | Y | Shared | ♒ |
| *phi-59* | T19B10.2 | 1.5 | 0 | 0.0 | ⬇ | 1 | Y | 121 | 1 | 6.5 | **＋** | Y | Shared | ♒ |
| *pod-2a* | W09B6.1 | 3.0 | 4 | 3.9 | – | 2 | Y | 324 | 1 | 2.3 | – | N | NHR-23 | ♒ |
| *ptr-4* | C45B2.7 | 4.5 | 4 | 2.6 | ⬇ | 2 | Y | 221 | 2 | 6.9 | – | N | NHR-23 | ♒ |
| *ptr-23* | ZK270.1 | 2.0 | 0 | 0.0 | – | 2 | N | 311 | 3 | 7.3 | **＋** | Y | *let-7s* | ♒ |
| *qua-1* | T05C12.10 | 5.7 | 3 | 1.5 | ⬇ | 4 | Y | 340 | 1 | 2.2 | **＋** | Y | Shared | ♒ |
|  | E03H4.8 | 3.4 | 1 | 0.9 | – | 0 | N | 307 | 1 | 2.5 | – | N | **–** | ♒ |
|  | T19A5.3 | 4.9 | 2 | 1.2 | ⬇ | 2 | Y | 347 | 1 | 2.2 | **＋** | Y | Shared | ♒ |
|  | Y47D3B.1 | 4.9 | 3 | 1.8 | – | 1 | Y | 98 | 1 | 8.1 | – | N | NHR-23 | ♒ |
| ***Extracellular Matrix Proteins and Receptors*** | | | | | | | | | | | | | | |
| *adt-2* | F08C6.1 | 7.6 | 7 | 2.7 | – | 6 | Y | 621 | 1 | 1.2 | **＋** | Y | Shared | ♒ |
| *bli-5* | F45G2.5 | 1.4 | 0 | 0.0 | – | 0 | N | 300 | 1 | 2.5 | **＋** | Y | *let-7s* | ♒ |
| *bus-8* | T23F2.1 | 3.6 | 0 | 0.0 | ⬇ | 0 | N | 453 | 2 | 3.3 | – | N | **–** | ♒ |
| *clc-1* | C09F12.1 | 5.4 | 0 | 0.0 | – | 0 | N | 101 | 1 | 7.8 | – | N | **–** | ♒ |
| *col-12* | F15H10.1 | 0.7 | 0 | 0.0 | – | 0 | N | 101 | 1 | 7.8 | **＋** | Y | *let-7s* | ♒ |
| *dpy-13* | F30B5.1 | 3.8 | 5 | 3.8 | – | 4 | Y | 63 | 2 | 26.1 | **＋** | Y | Shared | ♒ |
| *dpy-17* | F54D8.1 | 0.4 | 1 | 7.3 | – | 0 | N | 54 | 1 | 15.5 | – | N | **–** | ♒ |
| *dpy-4* | Y41E3.2 | 2.4 | 0 | 0.0 | – | 1 | N | 102 | 1 | 7.8 | **＋** | Y | *let-7s* | ♒ |
| *dpy-5* | F27C1.8 | 0.8 | 1 | 3.6 | ⬇ | 1 | Y | 39 | 0 | 0.0 | – | N | NHR-23 | ♒ |
| *dpy-7* | F46C8.6 | 0.7 | 2 | 8.3 | ⬇ | 1 | Y | 236 | 1 | 3.2 | – | N | NHR-23 | ♒ |
| *fbn-1* | ZK783.1 | 8.3 | 12 | 4.2 | ⬇ | 6 | Y | 457 | 1 | 1.7 | **＋** | Y | Shared | ♒ |
| *ina-1* | Y116A8A.9 | 8.0 | 8 | 2.9 | – | 2 | Y | 261 | 1 | 2.9 | **＋** | Y | Shared | ♒ |
| *mam-1* | ZC13.3 | 3.8 | 3 | 2.3 | – | 0 | N | 243 | 0 | 0.0 | – | N | **–** | ♒ |
| *mlt-10* | C09E8.3 | 8.6 | 4 | 1.3 | ⬇ | 1 | Y | 139 | 2 | 11.2 | **＋** | Y | Shared | ♒ |
| *mlt-11* | W01F3.3 | 5.2 | 11 | 6.1 | ⬇ | 4 | Y | 353 | 1 | 2.1 | **＋** | Y | Shared | ♒ |
| *mlt-7* | ZK430.8 | 11.5 | 15 | 3.8 | – | 1 | Y | 317 | 2 | 4.8 | **＋** | Y | Shared | ♒ |
| *mlt-9* | F09B12.1 | 3.5 | 1 | 0.8 | ⬇ | 5 | Y | 313 | 1 | 2.4 | – | N | NHR-23 | ♒ |
| *mup-4* | K07D8.1 | 5.8 | 5 | 2.5 | – | 2 | Y | 394 | 0 | 0.0 | – | N | NHR-23 | ♒ |
| *nas-36* | C26C6.3 | 1.1 | 1 | 2.6 | ⬇ | 0 | Y | 327 | 5 | 11.6 | **＋** | Y | Shared | ♒ |
| *nas-37* | C17G1.6 | 3.6 | 7 | 5.6 | ⬇ | 2 | Y | 240 | 4 | 12.7 | – | N | NHR-23 | ♒ |
| *noah-1* | C34G6.6 | 7.9 | 9 | 3.3 | ⬇ | 5 | Y | 550 | 1 | 1.4 | **＋** | Y | Shared | ♒ |
| *noah-2* | F52B11.3 | 8.9 | 3 | 1.0 | ⬇ | 6 | Y | 316 | 2 | 4.8 | **＋** | Y | Shared | ♒ |
| *pan-1* | M88.6 | 2.5 | 2 | 2.3 | – | 2 | Y | 393 | 2 | 3.8 | **＋** | Y | Shared | ♒ |
| *pat-2* | F54F2.1 | 4.0 | 4 | 2.9 | – | 2 | Y | 292 | 2 | 5.2 | **＋** | Y | Shared | ♒ |
| *rol-6* | T01B7.7 | 3.4 | 2 | 1.7 | ⬇ | 4 | Y | 117 | 1 | 6.7 | **＋** | Y | Shared | ♒ |
| *pat-3* | ZK1058.2 | 5.0 | 0 | 0.0 | – | 2 | N | 400 | 1 | 1.9 | **＋** | Y | *let-7s* | ♒ |
| ***Cytoskeletal Components*** | | | | | | | | | | | | | | |
| *ifa-2* | W10G6.3 | 1.7 | 1 | 1.7 | – | 1 | Y | 186 | 2 | 8.3 | – | N | Shared | ♒ |
| *ifc-2* | M6.1 | 3.0 | 0 | 0.0 | – | 0 | N | 536 | 2 | 2.8 | **＋** | Y | *let-7s* | ♒ |
| *nmy-2* | F20G4.3 | 1.8 | 6 | 9.7 | – | 1 | Y | 448 | 2 | 3.4 | **＋** | Y | Shared | ♒ |
| ***Genes linked to the molting cycle whose expression is not known to oscillate*** | | | | | | | | | | | | | | |
| *daf-9* | T13C5.1 | 1.1 | 0 | 0.0 | ⬇ | 1 | Y | 214 | 2 | 7.2 | **＋** | Y | Shared | **–** |
| *daf-12* | F11A1.3 | 17.0 | 12 | 2.0 | – | 7 | Y | 1393 | 5 | 2.7 | **＋** | Y | Shared | **–** |
| *gei-8* | C14B9.6 | 1.8 | 1 | 1.6 | – | 3 | Y | 449 | 4 | 6.7 | **＋** | Y | Shared | **–** |
| *let-767* | C56G2.6 | 0.6 | 1 | 4.8 | – | 1 | Y | 87 | 1 | 9.2 | **＋** | Y | Shared | **–** |
| *lin-3* | F36H1.4 | 5.5 | 5 | 1.9 | – | 0 | N | 442 | 5 | 8.5 | **＋** | Y | *let-7s* | **–** |
| *nhr-67* | C08F8.8 | 5.5 | 5 | 2.6 | – | 0 | N | 241 | 3 | 9.5 | – | N | **–** | **–** |
| *skn-1* | T19E7.2 | 5.1 | 2 | 1.1 | – | 2 | Y | 677 | 1 | 1.1 | **＋** | Y | Shared | **–** |
| ***Non-CCGs (Randomly Selected)*** | | | | | | | | | | | | | | |
| *acs-13* | Y65B4BL.5 | 4.9 | 3 | 1.8 | – | 2 | Y | 424 | 1 | 1.8 | ＋ | Y | Shared | **–** |
| *ced-8* | F08F1.5 | 0.7 | 0 | 0.0 | – | 0 | N | 85 | 0 | 0.0 | – | N | **–** | **–** |
| *cyp-33C12* | Y5H2B.6 | 1.5 | 0 | 0.0 | – | 0 | N | 148 | 0 | 0.0 | – | N | **–** | **–** |
| *ech-5* | F56B3.5 | 0.5 | 0 | 0.0 | – | 1 | N | 602 | 1 | 1.2 | – | N | **–** | **–** |
| *map-2* | Y116A8A.9 | 1.5 | 1 | 1.9 | – | 0 | N | 274 | 2 | 5.6 | – | N | **–** | **–** |
| *mpst-7* | R186.6 | 1.0 | 0 | 0.0 | – | 1 | N | 84 | 0 | 0.0 | ＋ | N | **–** | **–** |
| *nhr-176* | F14H3.11 | 0.2 | 0 | 0.0 | – | 0 | N | 54 | 1 | 15.5 | – | N | **–** | **–** |
| *nlp-37* | F48B9.4 | 2.9 | 3 | 3.0 | – | 0 | N | 302 | 2 | 5.0 | – | N | **–** | **–** |
| *nuo-2* | T10E9.7 | 0.2 | 0 | 0.0 | – | 1 | N | 109 | 1 | 14.5 | – | N | **–** | **–** |
| *srz-10* | ZK1037.11 | 1.1 | 1 | 2.6 | – | 0 | N | 16 | 0 | 0.0 | – | N | **–** | **–** |
| *ttll-12* | D2013.9 | 0.1 | 0 | 0.0 | – | 0 | N | 175 | 1 | 4.4 | ＋ | Y | *let-7s* | **–** |
| *unc-112* | C47E8.7 | 2.8 | 1 | 1.1 | – | 1 | Y | 295 | 1 | 2.6 | ＋ | Y | Shared | **–** |
| *viln-1* | C10H11.1 | 7.0 | 3 | 1.1 | – | 0 | N | 119 | 2 | 13.2 | – | N | **–** | **–** |
|  | C01G6.9 | 0.1 | 0 | 0.0 | – | 0 | N | 76 | 1 | 10.6 | – | N | **–** | – |
|  | F44E5.5 | 0.4 | 0 | 0.0 | – | 1 | N | 39 | 0 | 0.0 | – | N | **–** | **–** |
|  | R10E8.6 | 1.3 | 0 | 0.0 | – | 0 | N | 31 | 0 | 0.0 | – | N | **–** | **–** |
|  | R12B2.2 | 0.5 | 0 | 0.0 | – | 0 | N | 115 | 0 | 0.0 | – | N | **–** | **–** |
|  | T06D4.1 | 2.3 | 3 | 3.8 | – | 0 | N | 234 | 0 | 0.0 | – | N | **–** | **–** |
|  | W02D7.3 | 2.0 | 1 | 1.3 | – | 0 | N | 78 | 0 | 0.0 | – | N | **–** | **–** |
|  | Y53C10A.6 | 6.3 | 0 | 0.0 | – | 0 | N | 201 | 2 | 7.6 | – | N | **–** | **–** |
