# Supplementary material for: Feedback between a retinoid-related nuclear receptor and the let-7 microRNAs controls the pace and number of molting cycles in C. elegans
Source: eLife. 2022 Aug 15;11:e80010. doi: 10.7554/eLife.80010 (PMC9377799; doi:10.7554/eLife.80010)
Supplement: Supplementary file 5. — DNA or RNA sequences appear in the first column. For primers and gene blocks used to construct a particular bicistronic reporter for cis-regulatory elements in a 3′ UTR of interest, the resulting plasmid and corresponding extrachromosomal array are identified in the ‘application’ column. All seven reporters and respective transgenic strains of C. elegans are further described in The Key Resources Table. [file elife-80010-supp5.docx]

**Supplementary File 5. Oligonucleotides used in this study**

| **PCR Primer** | **Nucleotide Sequence (5' to 3')** | **Application** |
| --- | --- | --- |
| HM01 | GAAGAACGCCTCACCGAAGGAAGGAAGCATGCGGGATTGGCCAAAGGACCCAAAGGTATGTTTCGAATGATACTAACATAACATAGAACATTTTCAGGAGGACCCTTGGAGGGTAGAAAAAATGGTGAGCAAGGGCGAGGAGGTCATCAAAG | Construction of the bicistronic reporter for *cis*-regulatory elements in the 3' UTR of *unc-54* (pHR011 and *aaaEx97*) |
| *SL2::GFP:: unc-54*  cassette | GCTGTCTCATCCTACtttcacctagttaactgcttgtcttaaaatctatgcttctctttagtatctaaaattttcctagaagcttacaagtatataaatggtctcttctcaataaaggttgtatatttattcatcttattgaatctgccatttcctcgtttttgcgagtttatataccttccaattttctttctattgtattttcaacttctaattttaattcagggaaactgctgTACCGGTAGAAAAAATGAGTAAAGGAGAAGAACTTTTCACTGGAGTTGTCCCAATTCTTGTTGAATTAGATGGTGATGTTAATGGGCACAAATTTTCTGTCAGTGGAGAGGGTGAAGGTGATGCAACATACGGAAAACTTACCCTTAAATTTATTTGCACTACTGGAAAACTACCTGTTCCATGGGTAAGTTTAAACATATATATACTAACTAACCCTGATTATTTAAATTTTCAGCCAACACTTGTCACTACTTTCTGTTATGGTGTTCAATGCTTCTCGAGATACCCAGATCATATGAAACGGCATGACTTTTTCAAGAGTGCCATGCCCGAAGGTTATGTACAGGAAAGAACTATATTTTTCAAAGATGACGGGAACTACAAGACACGTAAGTTTAAACAGTTCGGTACTAACTAACCATACATATTTAAATTTTCAGGTGCTGAAGTCAAGTTTGAAGGTGATACCCTTGTTAATAGAATCGAGTTAAAAGGTATTGATTTTAAAGAAGATGGAAACATTCTTGGACACAAATTGGAATACAACTATAACTCACACAATGTATACATCATGGCAGACAAACAAAAGAATGGAATCAAAGTTGTAAGTTTAAACATGATTTTACTAACTAACTAATCTGATTTAAATTTTCAGAACTTCAAAATTAGACACAACATTGAAGATGGAAGCGTTCAACTAGCAGACCATTATCAACAAAATACTCCAATTGGCGATGGCCCTGTCCTTTTACCAGACAACCATTACCTGTCCACACAATCTGCCCTTTCGAAAGATCCCAACGAAAAGAGAGACCACATGGTCCTTCTTGAGTTTGTAACAGCTGCTGGGATTACACATGGCATGGATGAACTATACAAATAGgagctcCGCATCGGCCGCTGTCATCAGATCGCCATCTCGCGCCCGTGCCTCTGACTTCTAAGTCCAATTACTCTTCAACATCCCTACATGCTCTTTCTCCCTGTGCTCCCACCCCCTATTTTTGTTATTATCAAAAAAACTTCTTCTTAATTTCTTTGTTTTTTAGCTTCTTTTAAGTCACCTCTAACAATGAAATTGTGTAGATTCAAAAATAGAATTAATTCGTAATAAAAAGTCGAAAAAAATTGTGCTCCCTCCCCCCATTAATAATAATTCTATCCCAAAATCTACACAATGTTCTGTGTACACTTCTTATGTTTTTTTTACTTCTGATAAATTTTTTTTGAAACATCATAGAAAAAACCGCACACAAAATACCTTATCATATGTTACGTTTCAGTTTATGACCGCAATTTTTATTTCTTCGCACGTCTGGGCCTCTCATGACGTCAAATCATGCTCATCGTGAAAAAGTTTTGGAGTATTTTTGGAATTTTTCAATCAAGTGAAAGTTTATGAAATTAATTTTCCTGCTTTTGCTTTTTGGGGGTTTCCCCTATTGTTTGTCAAGAGTTTCGAGGACGGCGTTTTTCTTGCTAAAATCACAAGTATTGATGAGCACGATGCAAGAAAGATCGGAAGAAGGTTTGGGTTTGAGGCTCAGTGGAAGGTGAGTAGAAGTTGATAATTTGAAAGTGGAGTAGTGTCTATGGGGTTTTTGCCTTAAATGACAGAATACATTCCCAATATACCAAACATAACTGTTTCC | Construction of the bicistronic reporter for *cis*-regulatory elements in the 3' UTR of *unc-54* (pHR011 and *aaaEx97*) |
| HM04 | ATGGTGAGCAAGGGCGAGG | Construction of pHR011 and *aaaEx97* |
| HM27 | GCGGCCGCTTACTTGTACAGCTCGTCC |  |
| HM28 | GGACGAGCTGTACAAGTAAGCGGCCGCGTCCAATTACTCTTCAACATCCC |  |
| HM37 | GGTACCATGGTATTGAGCTGTCTCATCC |  |
| HM29 | CCGCGCACATTTCCCCGAAAAGTGCCACGGTACCCAAAAAAATTTATCAGAAG |  |
| HM32 | GTGGCACTTTTCGGGGAAATG |  |
| HM34 | CCTTTTCTGTACATGTCCTGGCCGGCCGGCCAGCAAAAGGCCAGGAACC |  |
| RA31 | GATGGCCGGCCTGATCGAAAGTCTCTCCGG |  |
| RA32 | CTAGTGATATCCATTTATCTGGAACAAAATGTAAG |  |
| RA101 | GGATCCCTGAATCCATATATCATC | Construction of *nhr-23* 3' UTR reporter (pHR017 and *aaaEx129*) |
| RA102 | GGTACCGAGACGTTTTATCACTG |  |
| RA190 | GGATCCACACTTTCTTCTTGCTCTTTACC | Construction of *lin-41* 3' UTR reporter (pHR023 and *aaaEx146*) |
| RA191 | GGTACCAATTTCGCAGTGAAATTTGCG |  |
| RA169 | TTAAAACTCGTATCATTCCAGTGTCTGC | Deletion of LCS from *nhr-23* 3' UTR reporter (pHR021 and *aaaEx131*) |
| RA170 | TTAATAAAATAAAAATTAGTGCGCCTAGAAATCC |  |
| RA171 | TTTGATCCAACCATTTTCTCGTTTATGG | Deletion of nucleotides 623-646 from *nhr-23* 3' UTR reporter (pHR022 and *aaaEx165*) |
| RA172 | GCAGACACTGGAATGATACGAGTTTTAA |  |
| RA184 | TTCTTTCTCTCCTTTTCCTGTTTTTAAAG | Deletion of nucleotides 227-249 from *nhr-23* 3' UTR reporter (pHR026 and *aaaEx166*) |
| RA185 | GACTACAATTATTTTTCTATTAATTTTCTG |  |
| RA168 | AACTATTGATGATATATGGATTCAGGGATCC | Deletion of nucleotides 26-42 from *nhr-23* 3' UTR reporter (pHR020 and *aaaEx130*) |
| RA167 | CCCTATCCCCGTCCATGAATC |  |
| RA227 | CACGGGTACAAAACCACAAATTTCC | Genotype *nhr-23(aaa20*) *I* |
| RA228 | GCGACCACTACACCATAAACG |  |
| RA272 | AGATGAGATGACTAATGAAAGTCCTCG | Genotype *dpy-10 II* |
| RA273 | AGTGAAGAAAGTCCTGCCTTATCC |  |
| RA202 | GGTGACAGCCCACTTGGTGCC | Genotype *let-7(n2853) X* |
| RA203 | TCCTTCTAAATTCGTCTAGGCGTCG |  |
| RA277 | GGGATAAATGATAAAATGATAACG | Genotype *nDf51 V* |
| RA278 | GGCCGAAAGGCTTCTTACAC |  |
| RA246 | GCTCAATTCTTGGAGCCAGC | Genotype *mir-84(n4037) X* |
| RA247 | GATTTTCTGCTCCGACAGATTAACATG |  |
| RA173 | TGCCAGACGGCATTCCCTAG | Genotype *let-7(mg279) X* |
| RA174 | AATCAAGTGTGCACTGACCACTC |  |
| RA109 | GCAACGGGAAGCTCTGTTACAGG | Genotype *mir-84(tm1304) X* |
| RA110 | GTTCCTCCATTCGACCATAAAGCC |  |
| oHG206 | GCTCTTCAAAACTTCCGAATGTCTG | Genotype *nhr-23(xk22) I* |
| oHG207 | AATAACCGGAGGAAACGAGATTCAT |  |
| oHG280 | CACTGCGTGACACCCGATTAA | Genotype *let-7(xk39) X*, *let-7(xk41) X, let-7(xk42) X, let-7(xk43) X, let-7(xk44) X* |
| oHG281 | TACATGCCCATTTCAAATGTTTCTT |  |
| oHG290 | CTCGAAGAACACAACATGTTATTTCAC | Sanger sequencing of *let-7(xk39) X*, *let-7(xk41) X, let-7(xk42) X, let-7(xk43) X, let-7(xk44) X* |
| oHG281 | TACATGCCCATTTCAAATGTTTCTT |  |
| oHG227 | ACTGCGTGACACCCGATTAAA | ChIP-qPCR for *let-7* promoter |
| oHG228 | CAAAATCCAGGTCACCGCAA |  |
| oHG235 | TCCATCTCTCTTGGAAACACAT | ChIP-qPCR for *col-19* promoter |
| oHG236 | ACACCTTCAAACCTAACCAGTGT |  |
| oHG294 | GCCTGACTCAGACTTCTCCATAGAT | ChIP-qPCR for *mir-84* promoter |
| oHG295 | AGAAGAGGAAAGGAAAAAAACAAGTTA |  |
| oHG298 | GCTCGGTGCCGTGTACTTTTATA | ChIP-qPCR for *mir-241* promoter |
| oHG299 | CCAACTTTCCATCTCTGTCGTCT |  |
| oHG300 | AAGCGGATCGAGGGAAAAGA | ChIP-qPCR for *mir-48* promoter |
| oHG301 | CCTCTCTAGTTCCTTCTGACTCTCTTG |  |
| oHG373 | GGACAGACTGTGGACATCCGA | ChIP-qPCR for *let-7* promoter in the *let-7(scRORE)* mutants |
| oHG374 | GCACGGAACCAACTTGCACT |  |
| oHG251 | CACCACCATCATCGCAAACC | ChIP-qPCR for *nhr-23* promoter, amplicon A |
| oHG252 | AACGGTACGGTATGCCTCC |  |
| oHG253 | CTGAGGGTCAGTGGTGTGAAA | ChIP-qPCR for *nhr-23* promoter*,* amplicon B |
| oHG254 | ACACAAAACACCTGCGTTCTC |  |
| oHG306 | CCCTTTCATGCACTATTCCGAGA | ChIP-qPCR for *lin-42* promoter, amplicon A |
| oHG307 | CCACCACCGCTAAACCTTTTG |  |
| oHG304 | GCGGAGACGCAGAGTACAG | ChIP-qPCR for *lin-42* promoter*,* amplicon B |
| oHG305 | TGCGAGACATGCCTACAGC |  |
| oHG193 | CAAGCAGGCGATTGGTGGA | qPCR primers for *pri-let-7* |
| oHG194 | GACGCAGCTTCGAAGAGTTCTGTC |  |
| oTH1269 | acgctcgtgatgagttcaag | qPCR primers for *eft-2* |
| oTH1270 | atttggtccagttccgtctg |  |
| **CRISPR** | **Nucleotide Sequence (5' to 3')** | **Application** |
| *dpy-10* crRNA | GCUACCAUAGGCACCACGAGGUUUUAGAGCUAUGCUGUUUUG | Edit the *dpy-10* locus |
| RA226 | CACTTGAACTTCAATACGGCAAGATGAGAATGACTGGAAACCGTACCGCATGCGGTGCCTATGGTAGCGGAGCTTCACATGGCTTCAGACCAACAGCCTAT | *dpy-10* ssODN |
| *nhr-23* crRNA | GAGUUUUAAAAGGCAAUAAAGUUUUAGAGCUAUGCUGUUUUG | Edit the 3' UTR of *nhr-23* |
| tracrRNA | AACAGCAUAGCAAGUUAAAAUAAGGCUAGUCCGUUAUCAACUUGAAAAAGUGGCACCGAGUCGGUGCUUUUUUU | Trans-activation of CAS9 |
| RA225 | CTGGATTTCTAGGCGCACTAATTTTTATTTTATTAATTAAAACTCGTATCATTCCAGTGTCTGCGCTTTAA | Repair template (ssODN) for excision of the LCS from 3' UTR of *nhr-23* |
| oHG202 crRNA | AUGAUAUAUGGAUUCAGUCA | Edit the final exon of *nhr-23* |
| oHG257 | TAGGGAAGGAGAGCATGGATAAAACTATTGATGATATATGGATTCAGTCATCCCTTGTCGTCGTCGTCCTTGTAGTCGATATCATGGTCCTTGTAATCTCCGTCGTGATCTTTATAGTCCGATCCCGATCCTCCTGAGCCTCCAGGCCGATCTGCAGTGAATAGCTCTTTGTAGAGGGCAGGAAGCTTTTCAG | Repair template (ssODN) for insertion of the coding sequence for 3xFLAG between the last coding codon and the stop codon of *nhr-23* |
| oHG278 crRNA | GGTATTTTATTGCGGTGACC | Edit RORE3 in the promoter of *let-7* |
| oHG291 | CTTTTGTTCCACTTTTGATGGTATTTTATTGCGGgtctacGGATTTTGCAACATGTGCATTCGAGGGTAAAGGAAG | Repair template (ssODN) for scrambling RORE3 in the promoter of *let-7* |
| oHG282 crRNA | CGCAGTGCTAGCCGTTGCAC | Edit RORE2 in the promoter of *let-7* |
| oHG292 | CAAAAAAACAGTGCAAGTTGGTTCCGTGCAAACAAgtctacGTGCAACGGCTAGCACTGCGTGACACCCGATTAAA | Repair template (ssODN) for scrambling RORE2 in the promoter of *let-7* |
| oHG287 crRNA | AAACTATCTAGGAGGGAACT | Edit RORE1 in the promoter of *let-7* |
| oHG293 | AGGAATTGAAAGTGGACAGACTGTGGACATCCGAGgcctaCAGTTCCCTCCTAGATAGTTTTTTTTCGCTTTCAA | Repair template (ssODN) for scrambling RORE1 in the promoter of *let-7* |
| oHG3 | TCCGAGgcctaCAGTTCCCTCCTAGATAGTTTTTTTTCGCTTTCAACTCCGCCCACAAAAAAACAGTGCAAGTTGGTTCCGTGCAAACAAgtctacGTGCAACGGCTAGCACTGCGTGACACCCGATTAAA | Repair template (ssODN) for scrambling RORE1 and RORE2 in the promoter of *let-7* |
